# Exclusive breastfeeding and associated factors among mothers in Debre Markos, Northwest Ethiopia: a cross-sectional study

**DOI:** 10.1186/s13006-014-0027-0

**Published:** 2015-01-20

**Authors:** Getnet Mekuria, Melkie Edris

**Affiliations:** College of Medicine and Health Sciences, Department of Public Health, Debre Markos University, Debre Markos, Ethiopia; Institute of Public Health, College of Medicine and Health Sciences, Department of Applied Human Nutrition, University of Gondar, Gondar, Ethiopia

## Abstract

**Background:**

Exclusive breastfeeding is the most widely known and effective intervention for preventing early-childhood deaths. Optimum breastfeeding practices can prevent 1.4 million deaths worldwide among children under five every year. The aim of this study was to assess the prevalence of exclusive breastfeeding and associated factors among mothers who have an infant less than six months old in Debre Markos, Northwest Ethiopia.

**Methods:**

A community based cross-sectional study was conducted from April 1 to 30, 2013. A simple random sampling technique was used from a list of all mothers who had an infant less than six months old obtained from the health extension workers (HEWs) registration book in all kebeles (neighbourhoods) of the city. A total of 423 mothers with infants less than six months old were included in this study. Data were collected using questionnaires administered at interview. Both bivariate and multivariate logistic regression analyses were carried out to identify factors associated with exclusive breastfeeding.

**Results:**

The prevalence of exclusive breastfeeding during the seven days before the survey was 60.8% (95% CI: 55.8%, 65.8%). Those mothers who were unemployed [AOR = 1.98 (1.21, 3.22)], received breastfeeding counseling during antenatal care (ANC) [AOR = 2.44 (1.53, 3.91)], received infant feeding counseling during postnatal care (PNC) [AOR = 5.03 (3.04, 8.31)], didn’t give prelacteal feeding [AOR = 3.44 (1.88, 6.33)] and had adequate knowledge about breastfeeding [AOR = 2.57 (1.57, 4.19)] were more likely to practice EBF than their counterparts.

**Conclusions:**

Although the prevalence of exclusive breastfeeding was lower in the study area than international recommendations, rates were higher than found in other studies. Recommendations for improving exclusive breastfeeding include better support for working mothers through extending maternal leave and establishing work-site day care centers for infants, expanding the urban health extension program so that more pregnant women and mothers can be taught about appropriate infant and young child feeding practices and how to express their milk, thereby increasing their breastfeeding knowledge.

## Background

Breast milk is a natural, renewable food that serves as a complete source of infant nutrition for the first six months of life. It has the appropriate balance of nutrients provided in a bio-available and easily digestible form, protecting both mothers and children against illnesses and diseases with unparalleled immunological and anti-inflammatory properties [1].

Exclusive breastfeeding is defined as feeding an infant with only breast milk and no additional food, water, or other liquids (with the exception of medicines and vitamins, if needed) during the first six months of life. Infants who are exclusively breastfed have less chance of becoming ill or dying from diarrhoea and other infections. In addition, they are less likely to acquire pneumonia, meningitis, and ear infections than non-breastfeed infants [2]. Exclusive breastfeeding is the most widely known and effective intervention for preventing early-childhood deaths. Optimum breastfeeding practices can prevent 1.4 million deaths worldwide among children under five every year [3]. Suboptimal breastfeeding contributes to 45% of neonatal infectious deaths, 30% of diarrheal deaths and 18% of acute respiratory deaths among children under five in developing countries [4]. It is also responsible for 10% of the disease burden in children younger than 5 years old [5].

Globally, fewer than 35% of infants are exclusively breastfed during the first four months of life [6]. In developing countries, just 37% of infants less than 6 months old are exclusively breastfed. In Africa, less than one third of infants under 6 months old are exclusively breastfed [7].

In Ethiopia approximately half (52%) of children less than six months old are exclusively breastfed. The practice of EBF at age 0–1 month is 70%, 55% at 2–3 months and 32% among 4–5 month old infants [8]. The Health Sector Development Program (HSDP) IV has set a target to increase exclusive breastfeeding from 49% to 70% in Ethiopia by the end of 2015 [9]. Therefore, this study is important to determine the prevalence of exclusive breastfeeding and to identify the factors that may act as barriers to mothers’ exclusively breastfeeding.

## Methods

### Study setting and participants

A community based cross-sectional study was conducted from April 1 to 30, 2013 in Debre Markos, located 265 km Northwest of Addis Ababa, Ethiopia. The town has seven kebeles, one Government hospital and three health centers. It has an estimated total population of 86,786, including 1,129 infants less than 6 months old.

The sample size was calculated by using a single population proportion formula with the following considerations; prevalence of exclusive breastfeeding practice (51.8%) from previous research conducted in Harar, Eastern Ethiopia [10], 95% confidence level, 5% margin of error and a 10% non-response rate. A total of 423 mothers were selected using a simple random sampling technique from a list of all mothers who had an infant less than six months old, obtained from the health extension workers registration book.

### Measurements

A questionnaire administered at interview was used to collect data from mothers. First, the English version of the questionnaire was prepared, translated to Amharic and then back to English by language translators in order to check for consistency. The questions were adapted from the Ethiopia Demographic and Health Survey (EDHS) 2011 and from other studies but modified accordingly.

A seven day self-recall method was used for assessing exclusive breastfeeding. Ten female diploma nurses were recruited for data collection and two Bachelor of Science nurses for supervision, with all attending a one-day training prior to data collection.

### Operational definitions

#### Exclusive breastfeeding

If an infant less than six months old took only breast milk and no additional food, water, or other liquids (with the exception of medicine and vitamins, if needed) during the seven days before the interview was conducted.

#### Predominant breastfeeding

If the infant’s predominant source of nourishment is breast milk but additionally took liquid foods including water, tea, cow/formula milk, juice, or salt/sugar solution during the seven days before the interview was conducted.

#### Partial breastfeeding

If the infant took breast milk and in addition soft foods like mashed potatoes/meat, fruits, porridge, egg, butter and liquids like cow/formula milk, water, tea, juice, or salt/sugar solution during the seven days before the interview was conducted.

#### Replacement feeding

If the infant took only soft foods like mashed potatoes/meat, fruits, porridge, egg, butter and liquids like cow/formula milk, water, tea, juice, or salt/sugar solution during the seven days before the interview was conducted.

#### Prelacteal feeding

If an infant during the first three days of life took something other than breast milk.

### Adequate knowledge about breastfeeding

Seven questions were asked to measure breastfeeding knowledge. If a mother could answer four or more questions correctly she was assessed as having adequate knowledge. The questions were adapted from previous similar unpublished thesis reports as well as the Infant and Young Child Feeding Strategy of Ethiopia [2].

### Statistical analysis

Data were checked for completeness and entered (double entry) into Epi Info version 3.5.3. Data were cleaned and coded using Epi Info and transferred to SPSS version 20 for analysis.

Descriptive statistics were used to characterize respondents using different variables of interest. First, bivariate logistic regression analysis was undertaken for each explanatory variable with the outcome variable (exclusive breastfeeding). Variables with a p-value ≤ 0.2 on bivariate logistic regression analysis were included in the multivariate logistic regression analysis. Statistical significance was determined using odds ratio with a 95% confidence interval. Statistical significance was declared if p-value was < 0.05.

Ethical approval was obtained from the Institutional Review Board of the Institute of Public Health, College of Medicine and Health Sciences, University of Gondar. A supporting letter was also provided by Debre Markos administration health office to conduct the research. In addition, informed verbal consent was obtained from the respondents before interviewing. Respondents were told about the aim of the study and confidentiality of the information they provided. Participants were informed that they had the right to withdraw from the study at any time. At the end of the interview mothers were informed about the benefits of breastfeeding for infants as well as for themselves and the risks of not breastfeeding.

## Results

### Socio-demographic characteristics

Of the 423 eligible mothers, 413 agreed to participate in the study, which made a response rate of 97.6%. Three hundred and sixty study participants were Amhara (87.2%), 36 (8.7%) Awi and 11 (4.1%) other ethnicities including Tigre and Oromo (Table 1). With regard to religion, the majority (83.8%) were orthodox Christian. The mean age of the mothers was 29.2 years (standard deviation, SD ± 4.9). The mean age of the infants was 3.4 months (SD ± 1.6).

**Table 1:** **Socio-demographic characteristics of mothers with infants aged less than six months, Debre Markos, Northwest Ethiopia, April 2013**

	**Category (n = 413)**	**Number**	**Percent**
Sex of infant	Male	219	53.0
	Female	194	47.0
Age of infant (days)	<60	79	19.1
	60-120	164	39.7
	121-179	170	41.2
Age of mother (years)	15-19	22	5.3
	20-24	76	18.4
	25-29	150	36.3
	30-34	104	25.2
	≥35	61	14.8
Religion of mother	Orthodox	346	83.8
	Muslim	24	5.8
	Catholic	20	4.8
	Protestant	23	5.6
Ethnicity of mother	Amhara	360	87.2
	Awi	36	8.7
	Other*	17	4.1
Education of mother	Not educated	98	23.7
	Educated	315	76.3
Employment status of mother	Unemployed	166	40.2
	Employed	247	59.8
Marital status of mother	Single	21	5.1
	Married	339	82.1
	Widowed	18	4.4
	Divorced	21	5.1
	Separated	14	3.4
Education of husband (n = 353)	Not educated	78	22.1
	Educated	275	77.9
Employment status of husband (n = 353)	Unemployed	21	5.9
	Employed	332	94.1
Type of family	Nuclear	280	67.8
	Extended	133	32.2
Monthly income**	<818	107	25.9
	818-1968	234	56.7
	>1968	72	17.4

*Tigre, Oromo.

**Ethiopian birr.

### Maternal and child health service utilization characteristics

The majority (78%) of mothers had antenatal care during their pregnancy, but only 47.9% received counseling concerning breastfeeding during their care (Table 2). The majority (72.6%) of the study participants had a postnatal care visit within 45 days of giving birth, and 67.3% were counseled about infant feeding at this visit.

**Table 2:** **Distribution of mothers with infants aged less than six months by maternal and child health service utilisation characteristics, Debre Markos, Northwest Ethiopia, April 2013**

**Variable**	**Category (n = 413)**	**Number**	**Percent**
Number of children	1	200	48.4
	2-3	182	44.1
	≥4	31	7.5
Birth order of infant	1	201	48.7
	2-3	178	43.1
	≥4	34	8.2
Antenatal care	Yes	322	78.0
	No	91	22.0
Breastfeeding counseling during antenatal care	Yes	198	47.9
	No	215	52.1
Place of delivery	Home	67	16.2
	Health institution	346	83.8
Mode of delivery	Normal/vaginal	379	91.8
	C/S	34	8.2
Postnatal care	Yes	300	72.6
	No	113	27.4
Infant feeding counseling during postnatal care	Yes	278	67.3
	No	135	32.7

### Breastfeeding information and knowledge

The majority (90.1%) of study participants had received breastfeeding information (Table 3). Over two thirds (61.7%) of mothers did not have adequate knowledge of breastfeeding. More than half (57.4%) of mothers knew that the first milk (colostrum) should be given to an infant (Table 4).

**Table 3:** **Distribution of mothers with infants aged less than six months by information access and breastfeeding knowledge characteristics, Debre Markos, Northwest Ethiopia, April 2013**

**Variable**	**Category (n = 413)**	**Number**	**Percent**
Media access	Radio only	52	12.6
	Television only	142	34.4
	Both radio and television	162	39.2
	Neither radio or television	57	13.8
Information ever heard about breastfeeding	Yes	372	90.1
	No	41	9.9
Source of information about breastfeeding*	Radio	105	25.4
	Television	215	52.1
	HEWs	207	50.1
	Other HWs	173	41.9
	VCHWs	36	8.7
	Family/friend/neighbor	71	17.2
Breastfeeding knowledge of mothers	Adequate	158	38.3
	Not adequate	255	61.7

*sum of percent >100 due to multiple response options.

HEWs – health extension workers.

HWs – health workers.

VCHWs – volunteer community health workers.

**Table 4:** **Breastfeeding knowledge of mothers with infants aged less than six months in Debre Markos Town, Northwest Ethiopia, April 2013 (n = 413)**

**No.**	**Question**	**Response**	**Number**	**Percent**
1.	Is breastfeeding important for child health?	Agree	349	84.5
		Don’t agree/don’t know	64	15.5
2.	Is breastfeeding important for maternal health?	Agree	91	22.0
		Don’t agree/don’t know	322	78.0
3.	Should an infant be put to breast immediately after birth?	Agree	259	62.7
		Don’t agree/don’t know	154	37.3
4.	Should the first milk/colostrum be given to an infant?	Agree	237	57.4
		Don’t agree/don’t know	176	42.6
5.	Is prelacteal feeding needed for an infant before starting breast milk?	Agree	262	63.4
		Don’t agree/don’t know	151	36.6
6.	Is breast milk alone without water and other liquids enough for an infant during the first 6 months of life?	Agree	147	35.6
		Don’t agree/don’t know	266	64.4
7.	Starting from 6 months, should an infant start complementary feeding and continued breastfeeding up to 2 years and beyond?	Agree	124	30.0
		Don’t agree/don’t know	289	70.0

### Breastfeeding practice

In this study all participants had initiated breastfeeding. Two hundred and fourteen respondents (51.8%) initiated breastfeeding immediately/within one hour of birth. The majority (75.8%) of infants were given prelacteal feeding. The prevalence of exclusive breastfeeding during the seven days before the interview was 60.8% (95% CI: 55.8%, 65.8%). Common reasons reported by mothers for not feeding breast milk only during the first six months of life includs; breastfeeding is not compatible with work, breastfeeding only is not sufficient for an infant, and they did not have enough milk (Table 5).

**Table 5:** **Distribution of mothers with infants aged less than six months by breastfeeding practices, Debre Markos, Northwest Ethiopia, April 2013**

**Variable**	**Category (n = 413)**	**Number**	**Percent**
Initiation of breastfeeding after birth	Immediately/within 1 hour	214	51.8
	After 1 hour	199	48.2
Colostrum feeding	Yes	259	62.7
	No	154	37.3
Prelacteal feeding	No	100	24.2
	Yes	313	75.8
Infant feeding during the seven days before interview	Exclusive breastfeeding	251	60.8
	Predominant breastfeeding	115	27.8
	Partial breastfeeding	38	9.2
	Replacement feeding	9	2.2
Reasons not feeding breast milk only (n = 162)	Not compatible with work	45	27.8
	Breastfeeding only is not sufficient	38	23.5
	Breast produce less milk	27	16.7
	Infant thirsty	22	13.6
	Mother ill/weak	13	8.0
	Breast/nipple problem	11	6.8
	Other	6	3.7

### Factors associated with exclusive breastfeeding

Hosmer-Lemeshow goodness-of-fit test (p = 0.617) was used to assess the fitness of the model. During the bivariate logistic regression analysis, educational status of the mother, employment status of mother, educational status of the husband, breastfeeding counseling during ANC, infant feeding counseling during PNC, colostrum feeding, prelacteal feeding and knowledge about breastfeeding were significantly associated with EBF.

During the multivariate logistic regression analysis; occupation of mother, breastfeeding counseling during ANC, infant feeding counseling during PNC, prelacteal feeding and knowledge about breastfeeding were significantly associated with EBF practice. But, educational status of the mother, educational status of the husband and colostrum feeding were not associated with EBF practice.

Unemployed mothers were 1.98 times more likely to exclusively breastfeed than employed mothers [AOR = 1.98 (1.21, 3.22)] (Table 6). Mothers who received counseling about breastfeeding during ANC were 2.44 times more likely to exclusively breastfed their infants compared to those who didn’t receive counseling [AOR = 2.44 (1.53, 3.91)]. Receiving counseling during PNC concerning infant feeding increased the likelihood of mothers feeding breast milk only by 5.03 times when compared to those who received no counseling during PNC [AOR = 5.03 (3.04, 8.31)]. Those mothers who didn’t give prelacteal feeding for their infant were 3.44 times more likely to exclusively breastfeed compared to those who gave prelacteal feeds[AOR = 3.44 (1.88, 6.33)]. Mothers who had adequate knowledge about breastfeeding were 2.57 times more likely to exclusively breastfeed their infants than those who didn’t have adequate knowledge on breastfeeding [AOR = 2.57 (1.57, 4.19)].

**Table 6:** **Bivariate and multivariate logistic regression analysis showing factors associated with EBF practice of mothers with infants aged <6 months in Debre Markos, Northwest Ethiopia, April 2013**

**Variable**	**Exclusive breastfeeding**		**Crude odds ratio (95% CI)**	**Adjusted odds ratio (95% CI)**
	**Yes (%)**	**No (%)**		
**Education of mother**				
Not educated	51 (12.3)	47 (11.4)	1	
Educated	200 (48.4)	115 (27.8)	1.6 (1.01, 2.53)	
**Employment status of mother**				
Unemployed	115 (27.8)	51 (12.3)	1.84 (1.22, 2.79)	1.98 (1.21, 3.22)*
Employed	136 (32.9)	111 (26.9)	1	1
**Education of husband**				
Not educated	36 (8.7)	42 (10.2)	1	
Educated	168 (40.7)	107 (25.9)	1.83 (1.10, 3.04)	
No husband	47 (11.4)	13 (3.1)	4.22 (1.98, 9.00)	
**Breastfeeding counseling during antenatal care**				
Yes	142 (34.4)	56 (13.6)	2.47 (1.64, 3.71)	2.44 (1.53, 3.91)*
No	109 (26.4)	106 (25.7)	1	1
**Infant feeding counseling during postnatal care**				
Yes	199 (48.2)	79 (19.1)	4.02 (2.61, 6.20)	5.03 (3.04, 8.31)*
No	52 (12.6)	83 (20.1)	1	1
**Colostrum feeding**				
Yes	168 (40.7)	91 (22.0)	1.58 (1.05, 2.37)	
No	83 (20.1)	71 (17.2)	1	
**Prelacteal feeding**				
No	82 (19.9)	18 (4.4)	3.88 (2.23, 6.77)	3.44 (1.88, 6.33)*
Yes	169 (40.9)	144 (34.9)	1	1
**Knowledge on breastfeeding**				
Adequate	119 (28.8)	39 (9.4)	2.84 (1.84, 4.40)	2.57 (1.57, 4.19)*
Not adequate	132 (32.0)	123 (29.8)	1	1

*P value <0.05 Backward stepwise logistic regression method.

## Discussion

The single most effective intervention for preventing child morbidity and mortality is practicing exclusive breastfeeding during the first six months of life [11]. In this study the prevalence of exclusive breastfeeding practice during the seven days before the interview was 60.8%, which almost meets the HSDP IV target of 70% by the end of 2015 [9]. The rate of exclusive breastfeeding practice was higher in this study than the national prevalence of 52% reported in the EDHS 2011 report [8].

The prevalence of EBF in this study is also higher than reported in previous international studies in the USA 16.8% [12], Brazil 29.8% [13], Klang Malaysia 32.8% [14], Peninsular Malaysia 43.1% [15], Tamil Nadu India 34% [16], Maharashtra India 36.84% [17], Al-Hassa Saudi Arabia 24.4% [18], Egypt 9.7% [19], Nigeria 31% [20], Western Tanzania 58% [21], Harar Eastern Ethiopia 51.8% [10], Arbaminch Southern Ethiopia 46.5% [22] and Mida-Woremo district of Northern Ethiopia 58.3% [23]. Some previous studies have identified higher rates of exclusive breastfeeding, including research at an urban health centre in Western India 61.5% [24], Zahedan Iran 98% [25] and the Goba district of South East Ethiopia 71.3% [26]. This variation might be due to methodological, time, socio-demographic, economic and cultural differences across areas.

Among the various socio-demographic factors assessed in this study only employment status of the mother was significantly associated with exclusive breastfeeding practice. Mothers who were unemployed were 1.98 times more likely to practice exclusive breastfeeding than employed mothers. This is in line with the study findings from Klang Malaysia [14], Peninsular Malaysia [15], Tamil Nadu India [16], Goba district [26], Arbaminch Ethiopia [22] and Mida-Woremo district Ethiopia [23]. This could be due to the fact that employed mothers may not have adequate time to feed breast milk to their infants during working hours.

In this study counseling about breastfeeding during ANC was significantly associated with exclusive breastfeeding for infants less than six months old. Those mothers who received counseling about breastfeeding during ANC were 2.44 times more likely to exclusively breastfed their infants compared to those who didn’t receive counseling. This is consistent with the study findings from Egypt [19], Nigeria [27], Harar, Ethiopia [10], Arbaminch, Ethiopia [22] and Mida-Woremo district, Ethiopia [23]. This could be due to the start of an urban health extension program which increases the number of women who receive ANC services including breastfeeding counseling that in turn improves breastfeeding knowledge of mothers.

Infant feeding counseling during PNC is another maternal and child health service factor that has a significant association with EBF. Those mothers who received counseling concerning infant feeding during PNC were 5.03 times more likely to feed breast milk only for their infants than those not counseled. This is in line with a study finding from an urban health centre of Western India [24]. This might be the result of increased expansion of health facilities with trained health professionals especially midwives who teach mothers appropriate infant and young child feeding practices.

Prelacteal feeding influences EBF adversely. It also shows a significant association with EBF in this study. Those mothers who didn’t give prelacteal feeds for their infant were 3.44 times more likely to exclusively breastfeed compared to those who gave prelacteal feeding. This is also consistent with a study finding from Saudi Arabia [18]. This could be explained by the fact that infants who were given prelacteal feeding may not adequately suckle their mothers’ breast which in turn decreases milk production. The decrease in milk production will lead mothers to introduce complementary foods early (before 6 months) for their infants.

Another factor that was shown to have a significant association with EBF is mothers’ breastfeeding knowledge. Mothers who had adequate knowledge about breastfeeding were 2.57 times more likely to exclusively breastfeed their infants than those who didn’t have adequate knowledge about breastfeeding. This has similarities with study findings from Nepal [28], Tunisia [29] and Tanzania [21]. This impact could be partly explained by mothers improved knowledge of the benefits of breastfeeding for themselves and their infants, as well as the risks of not breastfeeding, improving the likelihood mothers will breastfeed their infants even if alternatives are available.

## Conclusions

Although the prevalence of exclusive breastfeeding in the previous seven days was lower in the study area than international recommendations, rates were higher than found in other studies. Employment status of the mother, breastfeeding counseling during ANC, infant feeding counseling during PNC, prelacteal feeding and mothers’ knowledge about breastfeeding were independently and significantly associated with exclusive breastfeeding practice. Recommendations for improving EBF practices include better support for working mothers by extending maternal leave and establishing work-site day care centers for infants, expanding the urban health extension program so that more pregnant women and mothers can be taught about appropriate infant and young child feeding practices including how to express their milk if they are separated from their child.
